# Identification and Classification of Downy Mildew Severity Stages in Watermelon Utilizing Aerial and Ground Remote Sensing and Machine Learning

**DOI:** 10.3389/fpls.2022.791018

**Published:** 2022-05-20

**Authors:** Jaafar Abdulridha, Yiannis Ampatzidis, Jawwad Qureshi, Pamela Roberts

**Affiliations:** ^1^Department of Agricultural and Biological Engineering, Southwest Florida Research and Education Center, University of Florida, Immokalee, FL, United States; ^2^Department of Entomology and Nematology, Southwest Florida Research and Education Center, University of Florida, Immokalee, FL, United States; ^3^Department of Plant Pathology, Southwest Florida Research and Education Center, University of Florida, Immokalee, FL, United States

**Keywords:** artificial intelligence, hyperspectral imaging, plant disease, remote sensing, UAV

## Abstract

Remote sensing and machine learning (ML) could assist and support growers, stakeholders, and plant pathologists determine plant diseases resulting from viral, bacterial, and fungal infections. Spectral vegetation indices (VIs) have shown to be helpful for the indirect detection of plant diseases. The purpose of this study was to utilize ML models and identify VIs for the detection of downy mildew (DM) disease in watermelon in several disease severity (DS) stages, including low, medium (levels 1 and 2), high, and very high. Hyperspectral images of leaves were collected in the laboratory by a benchtop system (380–1,000 nm) and in the field by a UAV-based imaging system (380–1,000 nm). Two classification methods, multilayer perceptron (MLP) and decision tree (DT), were implemented to distinguish between healthy and DM-affected plants. The best classification rates were recorded by the MLP method; however, only 62.3% accuracy was observed at low disease severity. The classification accuracy increased when the disease severity increased (e.g., 86–90% for the laboratory analysis and 69–91% for the field analysis). The best wavelengths to differentiate between the DS stages were selected in the band of 531 nm, and 700–900 nm. The most significant VIs for DS detection were the chlorophyll green (Cl green), photochemical reflectance index (PRI), normalized phaeophytinization index (NPQI) for laboratory analysis, and the ratio analysis of reflectance spectral chlorophyll-a, b, and c (RARSa, RASRb, and RARSc) and the Cl green in the field analysis. Spectral VIs and ML could enhance disease detection and monitoring for precision agriculture applications.

## Introduction

Florida is the second-largest watermelon producer in the United States behind Texas. Based on the information from USDA Economic Research Service in 2017, more than 113,000 acres were cultivated throughout the United States, producing 40 million pounds of watermelon. The USA’s annual total watermelon budget was $578.8 million in 2016. Several diseases adversely impact watermelon production in Florida, and accurate disease identification is critical to implementing timely and effective management tactics. One of the significant diseases of watermelon is downy mildew (DM) caused by the fungal-like oomycete (*Pseudoperonospora cubensis*). Symptoms of DM are found on the leaves where the lesions begin as chlorotic (yellow) areas that become necrotic (brown/black) areas surrounded by a chlorotic halo. Under humid conditions, dense sporulation of the pathogen on the underside of the leaves within the lesions appears fuzzy. Severely affected leaves become crumpled and brown and may appear scorched. Downy mildew infection spreads quickly and, if left unchecked, can destroy an entire planting within days, hence the nickname “wildfire” for both the rapid disease development and scorched leaf appearance. Downy mildew does not affect stems or fruit directly; however, defoliation due to DM leaves fruit exposed to sunburning, making the fruit non-marketable. Therefore, successful field scouting for diseases would improve the yield production by implementing timely and effective management actions.

Because of the rapid and destructive occurrence of the disease, early detection and preventative fungicide applications are critical to its management. Non-destructive methods have been utilized as remote sensing tools for identifying and evaluating diseases occurring over a season (Immerzeel et al., 2008). Bagheri (2020) applied aerial multispectral imagery for the detection of fire blight infected pear trees by utilizing unmanned aerial vehicle (UAV), and several vegetation indices (VIs) (IPI, RDVI, MCARI1, MCARI2, TVI, MTVI1, MTVI2, TCARI, PSRI, and ARI) were evaluated for disease detection. The support vector machine (SVM) method was used to detect diseased trees with an accuracy of 95%. In another example, cotton root rot disease was detected by using UAV remote sensing and three automatic classification methods to delineate cotton root rot-contaminated areas (Wang et al., 2020). Ye et al. (2020) developed a technique for detecting and monitoring Fusarium wilt disease by utilizing UAV multispectral imagery. Eight VIs associated with pigment concentration and plant development changes were selected to determine the plants’ biophysical and biochemical characteristics during the disease progress development stages (Ye et al., 2020). Unmanned aerial vehicles can cover large crop areas by employing aerial photography to monitor the progress of a disease over time. Dense period sequence analysis can provide additional information on the timing of plant field changes and enhance the quality and accuracy of information derived from remote sensing (Woodcock et al., 2020).

One of the benefits of aerial imaging using UAVs is providing information on disease hot spots. Remote sensing (e.g., UAV-based hyperspectral imagery) can detect plants with diseases in asymptomatic and early disease development stages, which are critical for timely disease management (Hariharan et al., 2019). Abdulridha et al. (2019, 2020a) successfully detected different disease development stages of laurel wilt in avocado and bacterial and target spots in tomatoes with high classification accuracies utilizing remote sensing and machine learning. Lu et al. (2018) utilized spectral reflectance data to detect three different diseases in tomatoes, late blight, target spot, and bacterial spot, and several VIs were extracted to distinguish between healthy and diseased plants. Only a few studies utilized hyperspectral data for watermelon disease detection, which were conducted mainly in laboratories. Blazquez and Edwards (1986) utilized a spectroradiometer technique to measure the spectral reflectance of healthy watermelon and distinguish it from two diseases (Fusarium wilt and downy mildew). They found significant differences between the disease categories, especially in the NIR region (700–900 nm). Kalischuk et al. (2019) used UAV-based multispectral imaging and several VIs to detect watermelons infected with gummy stem blight, anthracnose, Fusarium wilt, Phytophthora fruit rot, Alternaria leaf spot, and cucurbit leaf crumple virus in the field.

However, all the benefits of remote sensing for disease detection are wasted (or squandered) if not timed correctly with early control management. If early disease detection is achieved and management practices are applied in time, limiting the disease spread throughout the field and minimizing economic losses are possible. One of the main goals of precision agriculture is to optimize fungicide and pesticide usage by detecting diseased areas (hotspots) and performing site-specific spraying. A sensor-based detection and mapping of stress symptoms in crops are required to accomplish spatially precise applications. Some recent studies have focused on sensor-based detection of pathogen infections in crops to implement site-specific fungicide applications (Abdulridha et al., 2020b,c). High-throughput phenotyping tools, the internet of things, and a smart environment can be used to observe the heterogeneity of crop vigor and could be helpful to optimize agricultural input usage through improved decisions on the spot and accurate timing and dose of chemical applications (West et al., 2003; Almalki et al., 2021; Saif et al., 2021; Alsamhi et al., 2022).

Early disease detection is key to limiting the spread, reducing the severity, and minimizing crop damage. Accurate disease identification at the beginning of an outbreak is essential for implementing effective management tactics. To the best of our knowledge, a high-throughput technique for detecting and monitoring DM severity stages in watermelon fields has not yet been developed. In this study, hyperspectral images were collected in the field (*via* UAVs) and in the laboratory (*via* a benchtop system) to (i) train ML models for the detection and monitoring of the DM for several DS stages in watermelon, and (ii) select the best bands and VIs to distinguish between a healthy and a DM-affected watermelon plant. To the best of our knowledge, we are the first to develop a UAV-based hyperspectral imaging technique to detect DM-affected watermelon plants in several disease severity (DS) stages.

## Materials and Methods

### Experimental Plot Design

The experiments were conducted at the University of Florida’s Southwest Florida Research and Education Center in Immokalee, FL, United States. Guidelines established by the University of Florida were followed for land preparation, fertility, irrigation, weed management, and insect control. The beds were 0.81 m wide with 3.66 m centers covered with black polyethylene film. Four-week-old watermelon transplants “Crimson Sweet” were planted on 9 March 2019 into the soil (Immokalee fine sand) in a complete randomized block treatment design with four replicates. Each plot consisted of 10 plants spaced 0.91 m apart within an 8.23 m row with 3.05 m between each plot. The plants were infected naturally by DM.

### Data Collection

**FIGURE 1:**
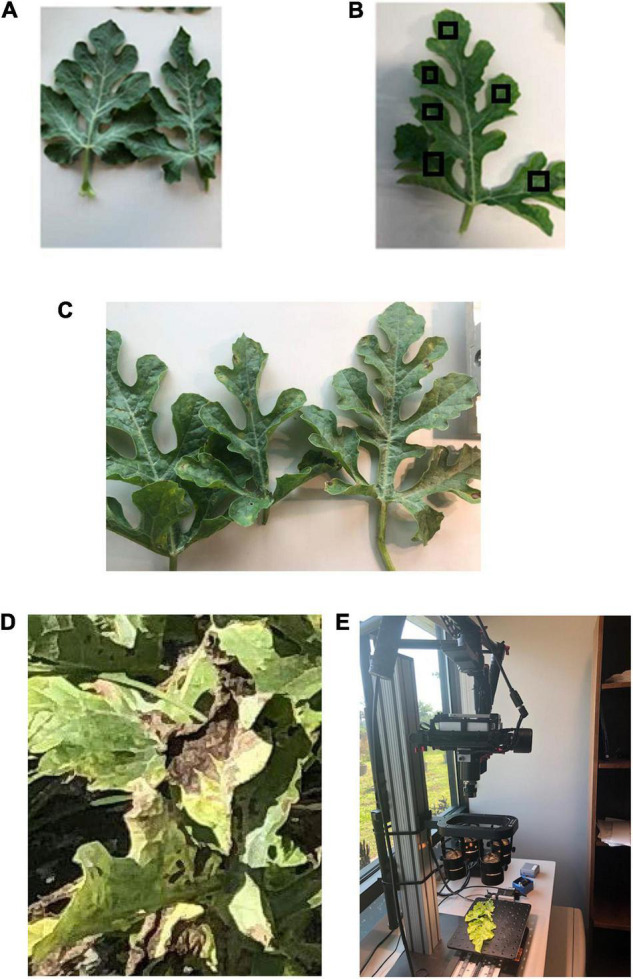
**(A)** Healthy watermelon leaves and downy mildew infected leaves in different severity stages (as examples): **(B)** low (this image includes examples of regions of interest, RoIs); **(C)** medium; and **(D)** high. **(E)** Hyperspectral data collection in the laboratory by a Pika L2 (Resonon Inc., Bozeman MT, United States) hyperspectral camera.

**FIGURE 2:**
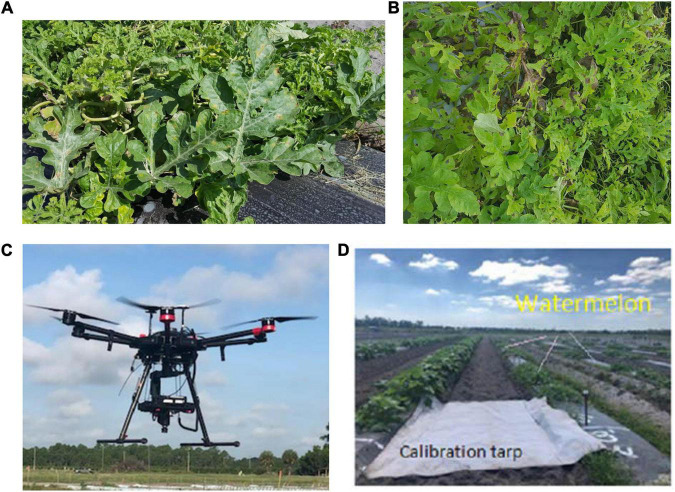
Downy mildew severity stages in the field: **(A)** low; **(B)** high; **(C)** UAV-based hyperspectral imaging system; and **(D)** a calibration tarp.

Healthy watermelon leaves were collected on April 10, 2019. After the first detection of DM, leaves were collected from DM-affected plants in five disease severities (DS; percentage of leaf tissue affected) stages. For the laboratory analysis, leaves were collected in the low DS stage (few lesions); then, from the medium 1 and 2, high and very high DS stages. The grading system of DM severity is as follows: the low DS had 5–10% severity, and as the disease progressed, the percentage of infection gradually increased in low to medium DS stages 1 and 2 (11–20% and 21–30% severity, respectively), and then the high and very high DS stages increased dramatically from 31 to 50% and 51 to 75% severity, respectively (Figure 1). In this study, spectral data were not collected when the DS was more than 75%, because the leaves were in very bad shape and desiccated. In the field (UAV-based) study, DS was categorized in two stages: low and high (Figure 2). The difference between the number of DS stages in the laboratory and field was due to the fact that, in the laboratory, each leaf was analyzed as a sample, and in the field, an entire plant was used as a sample. Hence, it was easier to quantify the DS stage in a single leaf than in an entire plant, which can include leaves in different DS stages.

### Data Collection and Analysis in the Laboratory

Spectral data were collected using a benchtop hyperspectral imaging system, Pika L 2.4 (Resonon Inc., Bozeman MT, United States) (Figure 1E). The Pika L 2.4 was equipped with a 23-mm lens, which has a spectral range of 380–1,030 nm, 15.3° field of view, and a spectral resolution of 2.1 nm. The same hyperspectral camera was utilized in the laboratory (Figure 1E) and field (Figure 2C) after changing lenses, which covered the same spectral range. Resonon’s hyperspectral imagers (RHI), known as push-broom imagers, are line-scan imagers, which have 281 spectral channels. The system is made up of a linear stage assembly, which is shifted by a stage motor. In the laboratory, controlled broadband halogen lighting sources were set up above the linear stage to produce ideal situations for conducting spectral scans. The hyperspectral imaging system was arranged in a way that the lens’ distance from the linear stage was 0.5 m. The lights were positioned at the same level as the lens on a parallel plane. All scans were performed using the Spectronon Pro (Resonon Inc., Bozeman, MT, United States) software, which was connected to the camera system using a USB cable. Before performing the scans of the leaves, dark current noise was removed using the software. Then, the camera was calibrated by using a white tile (reflectance reference), provided by the manufacturer, and placed under the same conditions as used for performing scans. The selection of the regions of interest (RoIs) (e.g., Figure 1B) was done manually by picking six spectral scan regions per leaf (10 leaves per DS stage) to prevent the occurrence of any bias. The total spectral scans (RoIs) selected for each DS stage was 60. Regions of interest were selected in such a way that they included both the affected and unaffected areas of leaf tissue. The pixel number of each spectral scan selected was between 800 and 900 pixels. The average of 60 spectral scans was used to form an overall spectral scan signature curve for each DS stage. The Spectronon Pro software, which is a post-processing data analysis software, was used to analyze the spectral data of each leaf scan. Several areas containing the symptomatic and non-symptomatic regions on the leaves were selected using the selection tool and the spectrum was generated. For the healthy and DM-affected plans (five DS stages), several random spots on leaves were selected and the average spectral reflectance was calculated and used to form the spectral signature curves.

### Data Collection and Analysis in the Field

Spectral data were collected in the field by using a UAV (Matrice 600 Pro, Hexacopter, DJI Inc., Shenzhen, China) and the same hyperspectral camera (Pika L 2.4). The UAV-based imaging system included (Figure 2C): (i) a Resonon Pika L 2.4 hyperspectral camera; (ii) a visible-NIR (V-NIR) objective lens for the Pika L camera with a focal length of 17 mm, field of view (FOV) of 17.6 degrees, and an instantaneous field of view (IFOV) of 0.71 mrad; (iii) a Global Navigation Satellite System (GNSS) (Tallysman 33-2710NM-00-3000, Tallysman Wireless Inc., Ontario, Canada)/Inertial Measurement Unit (IMU) (Ellipse N, SGB Systems S.A.S., France) flight control system for multirotor aircraft to record sensor position and orientation, and (iv) a Resonon hyperspectral data analysis software (Spectronon Pro, Resonon, Bozeman, MT, United States), which is capable of rectifying the GPS/IMU data using a georectification plugin. The data were collected at 30 m above the ground and a speed rate of 1.5 m s^–1^. In the produced map, the pixel size is a function of the working distance (distance between the camera lens and the scanning stage/field) and FOV. This value varies according to the flight parameters. In this study, it was around 35 mm per pixel. Gray tarp (Group VIII Technologies) was utilized to correct the data reflectance from radiance; the reflectance tarp was 36%. Radiometric calibration was performed by using a calibrated integrating sphere. The manufacturer took 100 lines of spectral data and built a radiometric calibration file that contains a lookup table with all combinations of integration times and frame rates. These data were used to convert raw camera data (digital numbers) to physical units of radiance in micro flicks. The Pika L 2.4 camera is a “pushbroom” or line-scan type imager that produces a 2-D image, where every pixel in the image contains a continuous reflectance spectrum. A calibration tarp was used to calibrate the data for various illumination conditions in the field (Figure 2D). The RoIs were randomly handpicked for each plant, and several spectral scans were done to cover the entire canopy. Each RoI contained four pixels, and four RoIs were selected for each plant. The total sample size for each DS stage was 20 plants. The RoIs were then transferred as a text file and processed using the SPSS software (SPSS 13.0, Inc., Chicago; Microsoft Corp., Redmond, WA, United States).

### Vegetation Indices

Vegetation indices could serve as indicators to identify DS stages based on any defectiveness in the functioning of plants such as physiochemical defects that affect photosynthesis, metabolic, and nutritional processes. The factors that are most affected by diseases and that could be measured are chlorophyll content, cell structure, cell sap, presence, and relative abundance of pigments concentration, water content, and carbon as expressed in the solar-reflected optical spectrum (400–2,500 nm) (Xiao et al., 2014). A neural network multilayer perceptron was performed to select the best VIs that could identify DM disease and its DS. For data analysis and evaluation of all VIs evaluated in this study (Table 1), the SPSS software was used. The correlation coefficient was another parameter that was utilized in this study to evaluate its VI’s performance in detecting DM severity stages.

**TABLE 1:** Spectral vegetation indices evaluated for downy mildew disease detection.

Ratio analysis of reflectance spectral chlorophyll-a (RARSa)	$R\,A\,R\,S\,a=\frac{R\,675}{R\,700}$	Chappelle et al., 1992
Ratio analysis of reflectance spectral chlorophyll b (RARSb)	$R\,A\,R\,S\,b=\frac{R\,675}{\left(R\,700\times R\,650\right)}$	Chappelle et al., 1992
Ratio analysis of reflectance spectra (RARSc)	$R\,A\,R\,S\,c=\frac{R\,760}{R\,500}$	Chappelle et al., 1992
Pigment specific simple ratio (PSSRa)	$P\,S\,S\,R\,a=\frac{R\,800}{R\,680}$	Blackburn, 1998
Normalized difference vegetation index 761 (NDVI 761)	N⁢D⁢V⁢I⁢ 761=(R⁢761-R⁢651)(R⁢761+R⁢651)	Raun et al., 2001
Green NDVI (GNDVI)	$G\,N\,D\,V\,I=\frac{\left(N\,I\,R\,850-G\,580\right)}{\left(N\,I\,R\,850+G\,580\right)}$	Gitelson and Merzlyak, 1996
Photochemical reflectance index (PRI)	$P\,R\,I=\frac{\left(R\,531-R\,570\right)}{\left(R\,531+R\,570\right)}$	Gamon et al., 1992
Simple ratio index (SR900)	$S\,R\,900=\frac{R\,900}{R\,650}$	Jordan, 1969
Water stress and canopy temperature (NWI 2)	$N\,W\,I\,2=\frac{R\,970-R\,850}{R\,970+R\,850}$	Babar et al., 2006
Structure insensitive pigment index (SIPI)	$S\,I\,P\,I=\frac{\left(R\,840-R\,450\right)}{\left(R\,840-R\,670\right)}$	Penuelas et al., 1995
Normalized phaeophytinization index (NPQI)	$N\,P\,Q\,I=\frac{\left(R\,415-R\,435\right)}{\left(R\,415-R\,435\right)}$	Barnes et al., 1992
Normalized difference vegetation index 761 (NDVI 761)	N⁢D⁢V⁢I⁢ 761=(R⁢761-R⁢651)(R⁢761+R⁢651)	Raun et al., 2001
Normalized difference vegetation index 850 (NDVI 850)	N⁢D⁢V⁢I⁢ 850=(R⁢850-R⁢651)(R⁢850+R⁢651)	Raun et al., 2001
Simple ratio index (SR850)	$S\,R\,850=\frac{R\,850}{R\,650}$	Jordan, 1969
Modified triangular vegetation index1 (MTVI 1)	*MTVI 1* = *1.2[1.2(1.2(R760-R580)—2.5(R650-R580)]*	Haboudane et al., 2004
Modified triangular vegetation index2 (MTVI 2)	M⁢T⁢V⁢I⁢ 2=1.5⁢[1.2⁢(R⁢760-R⁢580)-2.5⁢(R⁢650-R⁢580)]SQ[(2*R760+1)∧2-(6*R760-5*SQ(R650)-0.5]	Haboudane et al., 2004
Renormalized difference vegetation Index (RDVI)	$R\,D\,V\,I=\frac{\left(R\,761-R\,651\right)}{S\,Q\,\left(R\,761+R\,651\right)}$	Roujean and Breon, 1995
Triangle vegetation index (TVI)	*TVI* = 0.5[120*(R761-R581)-200(R651-R581)]	Broge and Leblanc, 2001
Red-edge vegetation stress index 1 (RVS1)	R⁢V⁢S⁢1=[(R⁢651+R⁢e⁢d⁢E⁢d⁢g⁢e⁢ 750)2]-R⁢e⁢d⁢E⁢d⁢g⁢e⁢ 733	Merton, 1998
Green vegetation (VI green)	$V\,I\,G\,r\,e\,e\,n=\frac{\left(R\,760-R\,651\right)}{\left(R\,760+R\,651\right)}$	Gitelson et al., 2002
Transform chlorophyll absorption in reflectance index (TCARI)	*TCARI* = *3[(R740-R651)-0.2(R740-R581) (R740/R651)]*	Haboudane et al., 2002
Water index (WI)	$W\,I=\frac{R\,900}{R\,970}$	Penuelas et al., 1997
Modified chlorophyll absorption in reflectance index (mCARI 1)	*mCARI 1* = 1.2[(2.5*R761-R651)-1.3(R761-R581)]	Haboudane et al., 2004
Anthocyanin reflectance index (ARI)	$A\,R\,I=\left(\frac{1}{R\,550}\right)-\left(\frac{1}{R\,700}\right)$	Gitelson et al., 2001
Chlorophyll green (*Chlgreen*)	$C\,h\,l\,g\,r\,e\,e\,n=\frac{\left(R\,760/R\,800\right)}{\left(R\,540/R\,560\right)}$	Gitelson et al., 2003
Chlorophyll index green (*ClgreenNIR*/*Green*)	$C\,l\,g\,r\,e\,e\,n\,N\,I\,R/G\,r\,e\,e\,n=\left(\frac{N\,I\,R}{G\,r\,e\,e\,n}\right)-1$	Gitelson et al., 2003
Chlorophyll index red edge (*Clrededge*)	$C\,l\,r\,e\,d\,e\,d\,g\,e=\left(\frac{R\,780}{R\,705}\right)-1$	Gitelson et al., 2003

### Classification Methods

#### Decision Tree

A decision tree (DT) is a non-parametric managed learning process used for organization and regression. The objective of DT is to produce a model that calculates the value of a goal variable by learning simple choice instructions deducted from data features. A decision tree has the capability of handling data measured on different rulers in the absence of any models for the proportion distributions of the data individually from the modules, elasticity, and capability to handle non-linear relationships among features and modules (Friedl and Brodley, 1997). The decision tree can be qualified rapidly and are quick in execution. It is a widely used technique in image processing for detecting several plant diseases. In this study, the classification was accrued between healthy and DM-affected watermelon plans in several DS stages. The dataset was split into 70% training and 30% testing.

#### Multilayer Perceptron

Multilayer perceptron (MLP), which is a deep artificial neural network, was applied to identify the difference between healthy and several DM disease severity stages. Similar to a neural network, MLP is a function of predictors, also called inputs, or independent variables that minimize the prediction error of target variables, also called outputs. These models can learn by example. Thus, when using a neural network, there is no need to program how the output is obtained for the given certain input; rather, a learning algorithm is used by the neural network to calculate the relationship between input and output, which is then utilized to predict output with the entered input values. The neural network creates a fitted model in an analytical form, where the parameters are weight, bias, and network topology. The multilayer perceptron is a fully connected multilayer feed-forward supervised learning network trained by the back-propagation algorithm to minimize a quadratic error criterion; no values are fed back to earlier layers. The multilayer perceptron is composed of an input layer, a hidden layer, and an output layer. The input and output layers are not weighted, and the transfer functions on the hidden layer nodes are radially symmetric. The full dataset was randomly split into two datasets by partitioning the active dataset into training (70%) and testing (30%) samples. After learning, the MPL model was run on the test set that provided an unbiased estimate of the generalization error.

## Results

### Laboratory-Based Analysis

**FIGURE 3:**
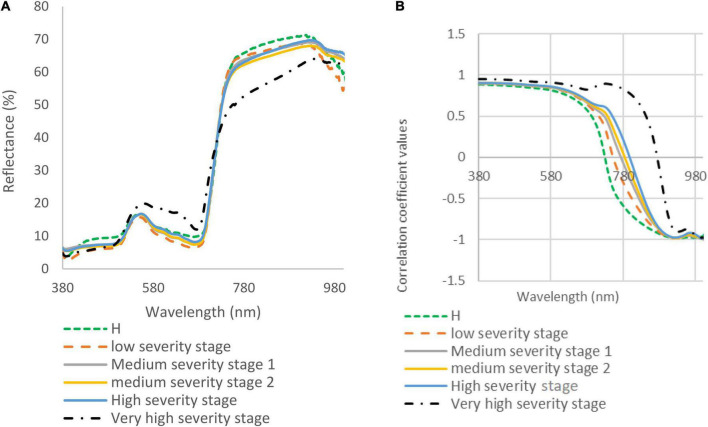
(A) Spectral reflectance signatures (collected in the laboratory) of downy mildew affected watermelon leaves in five disease severity (DS) stages; and (B) correlation coefficient for watermelon leaves in healthy (H) and five DS stages.

During the spectral data collection under optimal light and temperature conditions in the laboratory, the spectral reflectance of five DM disease severity stages was taken. The spectral signatures and correlation coefficient at different disease severities were measured and compared (Figure 3). The spectral reflectance of the very high DS stage showed a significant increase in the green and red range (450–650 nm), while other stages showed lower spectral reflectance in the visible range (380–700 nm). The spectral reflectance values of the very high DS stage were decreased in the NIR range, and the spectral reflectance in the red edge diverged from other stages. The leaves of watermelon in the low and medium DS stages had very few symptoms; therefore, there were only slight differences between the spectral reflectance for these stages. The spectral reflectance signature is strongly related to the severity of the expected DM pathogen symptom (Figure 3A). In the visible range (380–700 nm), there were not many differences in the correlation coefficient signature of all DS stages (Figure 3B). The signatures were identical until the red-edge range (700 nm), where it gradually showed differences between DS stages (especially the high and very high severity stages). It is obvious from Figure 3B that the correlation coefficient showed wide differences in the NIR range as the DS increased.

**FIGURE 4:**
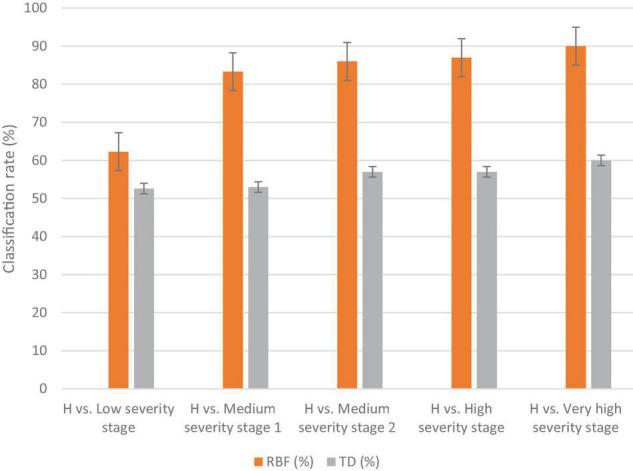
The classification results of the MLP and DT methods to distinguish healthy (H) against several disease severity stages of downy mildew disease in watermelon in the laboratory. The vertical lines on the columns are error bars.

The results of the classification varied based on disease severity, as in the low DS stage, the classification was lower than the other stages (e.g., 62.3 and 52.6% for MLP and DT, respectively). The classification rate increased as the severity of disease symptoms increased (Figure 4). The highest classification value of MLP was in the very high DS stage at 90%. The DT classification method had lower detection accuracies than the MLP classification method for all DS stages (Figure 4). Hence, the MLP method was selected as the best classification method for DM detection and DS stages classification. Therefore, it was used for the selection of the best wavebands and VIs for disease detection. The best wavebands for detecting the low and medium 1 DS stages were found on the red edge (711–722 nm), medium 2 and high DS stages were found at 1,007–1,020 nm, and the very high DS stage was found in red edge (759–761 nm). The best VIs, selected by using the MLP classification method, for low and medium DS stages 1 were the Cl green and PRI, respectively, while for medium 2, high, and very high DS stages were the NPQI (Table 2).

**TABLE 2:** Best wavebands and vegetation indices measured in the laboratory and field for detecting different disease severity stages of downy mildew.

Disease severity (DS) stages	The weight of best bands (95–100%)	Best vegetation indices
**Laboratory**		
Low	722 (100%), 711 (99%), 716 (98%), 709 (96%)	CI green
Medium 1	722 (100%), 720 (99%), 718 (99%), 716 (98%)	PRI
Medium 2	1,020 (100%), 1,014 (99%), 1,019 (98%), 1,016 (96%)	NPQI
High	1,010 (100%), 1,020 (99%), 1,014 (99%), 1,007 (97%)	NPQI
Very high	761 (100%), 759 (99%), 757 (99%), 763 (99%)	NPQI
**Field (UAV based)**		
Low	952 (100%), 956 (99%), 947 (98%), 965 (97%)	RARSc, RARSa
High	755 (100%), 771 (99%), 766 (99%), 780 (98%)	RARSb, CI green

### Field-Based Analysis

**FIGURE 5:**
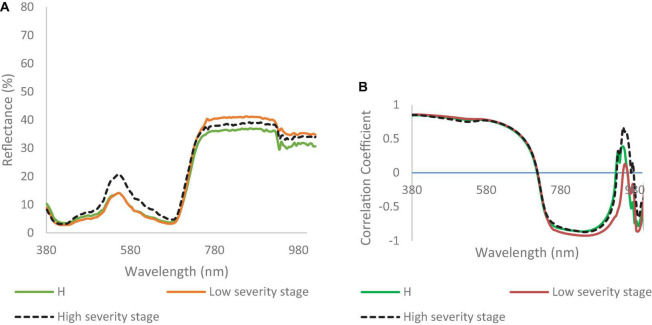
**(A)** Spectral reflectance signatures developed from hyperspectral imaging collected in the field; and **(B)** correlation coefficient for watermelon plant in healthy (H), low, and high downy mildew severity stages.

Figure 5A shows the UAV-based spectral signatures of healthy and DM-affected plants. In the visible range, the spectral reflectance values of healthy plants and DM-affected plans in a low DS stage were lower than in the high DS stage, which had a peak value of 20% at the green band. The spectral reflectance values of healthy plants were lower than the low and high DS stages in the NIR range. The spectral reflectance of the low DS stage was higher than the high DS stage, especially in the range of 750–915. Major differences cannot be seen in the red edge, where both DS stages had almost identical signatures. In the NIR range (700–1,000 nm), the spectral reflectance values of the low and high DS stages were higher than the spectral reflectance values of healthy plants.

The correlation coefficients for healthy, low, and high DS stages were almost identical in the visible range (Figure 5B). In the range of 750–1,000 nm, there were some differences recorded between the correlation coefficient values of the two DS stages (Figure 5B). Figure 5B shows differences between the low and high DS stages at 750 and 1,000 nm.

**FIGURE 6:**
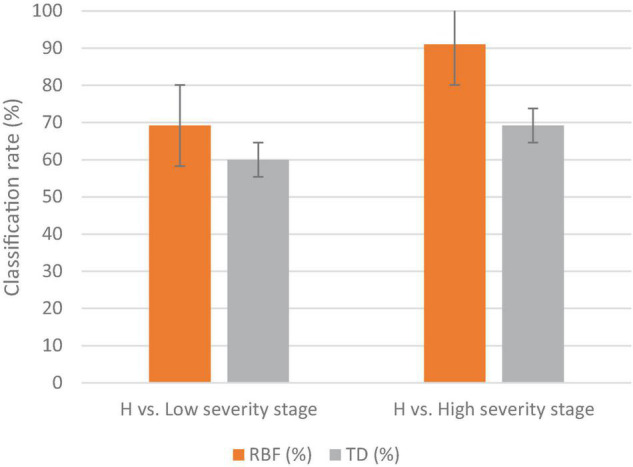
The classification results of the MLP and DT methodologies for detecting several disease severity stages of downy mildew in watermelon plants in the field against healthy plants (H). The vertical lines on the columns are error bars.

The MLP method gave a higher classification accuracy than the DT method; in the low DS stage, the classification accuracy of the MLP was 69%, while the classification accuracy of the DT was 60% (Figure 6). The highest classification accuracy was achieved in the high DS stage at 91% for MLP, while it was 69% for DT. The best wavebands for low DS stage classification were between 952 and 965 nm, while in the high DS stage the best wavebands were in red edge (755–780 nm). The best VIs for disease detection were the RARSc and RARSa for the low DS stage, and the RARSb and CI green for the high DS stage (Table 2).

## Discussion

Watermelon plants are very susceptible to diseases; therefore, several studies developed non-destructive and high-throughput techniques to detect diseases like anthracnose, leaf blight, and leaf spot (He et al., 2021). Blazquez and Edwards (1986) developed a laboratory-based technique to detect watermelon infected with Fusarium wilt, downy mildew, and watermelon mosaic. All three diseases had significant differences in the near infra-red range (700–900 nm). Kalischuk et al. (2019) applied UAV multispectral imaging to identify, using NDVI, gummy stem blight, anthracnose, Fusarium wilt, Phytophthora fruit rot, Alternaria leaf spot, and cucurbit leaf crumple disease. Disease incidence and severity ratings were significantly different between conventional scouting and UAV-assisted scouting. There is no other UAV-based system available for the detection and monitoring of DM in the field.

The difference between our results (aka, DS detection accuracy by the models, and best wavelengths and VIs for DS detection) in the laboratory and field can be explained by the different environmental conditions and data collection procedures. For example, in the laboratory, data are collected from single leaves at a close distance and with artificial light, in contrast to the field where data are collected by a UAV (30 m above ground) from entire plants. Hence, the results from the laboratory and field analysis cannot be directly compared.

### Spectral Reflectance Signatures

The DM-affected watermelon leaves showed varied spectral reflectance signatures for each DS stage (both in the laboratory and field). However, in the visible range, there were no significant differences between the healthy and the low DS stage (5–10% infection), which indicates that it is very difficult to distinguish among these stages with visual observation. For that reason, hyperspectral imaging and machine learning can be used for a more efficient and rapid early plant disease and severity detection (compared to visual detection methods).

Laboratory measurements showed that the spectral reflectance of healthy plants was higher than the other DS stages in the blue range. The main differences between healthy and infected plants in the spectral signatures, both in the laboratory and field measurements, were found in the high severity stage in the green, red edge, and NIR range (700–1,000), while in NIR, the spectral reflectance signature of healthy plants was higher than the other DS stages. The high DS stage had a unique signature that can be used to distinguish this stage from others (and healthy plants). Although we were able to visually observe the change of the colors of the leaves for some of the DS stages, not all had the same level of change.

In the field, the results did not show significant differences between the low and high DS stages in the visible range; only slight differences were observed. However, that minor differences in the spectral reflectance in the visible range can be still considered as an indication of color change in the leaves. The leaves and the plant canopy in the low DS stage showed very few visual symptoms, and the classification accuracy was low, both in the laboratory and field. As the chlorophyll content and water content decrease, the leaf cell damage increases (Barnawal et al., 2017), and that helps the classification methods to better classify the DS stages, especially in the medium and very high DS stages.

### Vegetation Indices

The common practice for selecting significant wavelengths for the DS detection of new VIs is by the correlation to a biochemical or biophysical trait, for example, chlorophyll a + b content leaf structure parameter, the water content, and so on (Gitelson and Merzlyak, 1996; Hatfield et al., 2008). Regularly, the fluctuation of spectral reflectance might guide to the detection of plants under stress without specifying or providing a description of what type of stress cause the damage to the plant (Carter and Miller, 1994; Gitelson and Merzlyak, 1994). For example, as was mentioned earlier, several factors could reduce the chlorophyll content, of which some are physiological or biochemical caused by DM severity and this reduction might influence the photosynthesis activity.

In both experimental conditions, the best vegetation indices (e.g., the PRI, this index is more related to the green range; and the CI green) were able to discriminate between healthy and DM-affected plants in the low and the high DS stages. The PRI, which is produced by normalizing 531 and 570 nm, basically relays on the green range. Any deficiency or disorder in chlorophyll will influence the PRI value; the PRI has increasingly been used as an indicator of photosynthetic efficiency (Garbulsky et al., 2011) and as an indicator of water stress (Suarez et al., 2008). One of the first changes in a DM-affected plant is the reduction of the chlorophyll concentration that affects the process of photosynthesis in the infected leaf, and some VIs associated with chlorophyll content could be used to detect these changes (Mandal et al., 2009; Bellow et al., 2013; Barnawal et al., 2017). Our findings suggest the same.

## Conclusion

The selected best spectral VIs resulted in high specificity and sensitivity for the detection and identification of downy mildew disease in different stages of severity. Lower classification results were achieved in the low DS stage, because of the minor changes in leaf composition (compared to a healthy plant). The highest classification results were obtained from the MLP method in high and very high DS stages (87–90%), while the DT method recorded lower classification results (compared to MLP) for all DS stages. Some VIs can be used for disease detection and classification of the DS stages. The use of hyperspectral imaging for identifying the most significant VIs to detect and identify several DS stages will further enhance the understanding and specificity of disease detection. Future work includes the development of a simple and inexpensive UAV-based sensor, based on previous research and developments, that only measures spectral reflectance at narrow bands (e.g., customized multispectral camera), centered at specific wavelengths for early DM detection in the field.

## Data Availability Statement

The raw data supporting the conclusions of this article will be made available by the authors, without undue reservation.

## Author Contributions

JA, YA, JQ, and PR conceived, designed, processed, analyzed, and interpreted the experiments. JA, YA, and PR acquired the data. JA and YA analyzed the data and prepared the manuscript. JQ and PR edited the manuscript. All authors contributed to the article and approved the submitted version.

## Author Disclaimer

The contents are solely the responsibility of the authors and do not necessarily represent the official views of the USDA.

## Conflict of Interest

The authors declare that the research was conducted in the absence of any commercial or financial relationships that could be construed as a potential conflict of interest.

## Publisher’s Note

All claims expressed in this article are solely those of the authors and do not necessarily represent those of their affiliated organizations, or those of the publisher, the editors and the reviewers. Any product that may be evaluated in this article, or claim that may be made by its manufacturer, is not guaranteed or endorsed by the publisher.
